# The lived experience of severe mental illness and long-term conditions: a qualitative exploration of service user, carer, and healthcare professional perspectives on self-managing co-existing mental and physical conditions

**DOI:** 10.1186/s12888-022-04117-5

**Published:** 2022-07-19

**Authors:** C. Carswell, J. V. E. Brown, J. Lister, R. A. Ajjan, S. L. Alderson, A. Balogun-Katung, S. Bellass, K. Double, S. Gilbody, C. E. Hewitt, R. I. G. Holt, R. Jacobs, I. Kellar, E. Peckham, D. Shiers, J. Taylor, N. Siddiqi, P. Coventry, on behalf of the DIAMONDS Research team

**Affiliations:** 1 Department of Health Sciences, University of York, York, UK; 2 Clinical and Population Sciences Department, Leeds institute for Cardiovascular and Metabolic Medicine, University of Leeds, Leeds, LS2 9JT UK; 3 Leeds Institute of Health, University of Leeds, Leeds, UK; 4 Population Health Sciences Institute, Newcastle University, Newcastle, UK; 5 Department of Sport and Exercise Sciences, Manchester Metropolitan University, Manchester, UK; 6 Bradford District Care NHS Foundation Trust, Bradford, UK; 7 Hull York Medical School, York, UK; 8 York Trials Unit, Department of Health Sciences, University of York, York, UK; 9 Human Development and Health, Faculty of Medicine, University of Southampton, Southampton, UK; 10 National Institute for Health Research Biomedical Research Centre, University Hospital Southampton NHS Foundation Trust, Southampton, UK; 11 Centre for Health Economics, University of York, York, UK; 12 School of Psychology, University of Leeds, Leeds, UK; 13 Psychosis Research Unit, Greater Manchester Mental Health NHS Trust, Manchester, UK; 14 Division of Psychology and Mental Health, University of Manchester, Manchester, UK; 15 School of Medicine, Keele University, Staffordshire, UK; 16 York Environmental Sustainability Institute, University of York, York, UK

**Keywords:** Severe mental illness, Mental health, Multimorbidity, Self-management, Qualitative research, Long-term conditions

## Abstract

**Background:**

People with severe mental illness (SMI), such as schizophrenia, have higher rates of physical long-term conditions (LTCs), poorer health outcomes, and shorter life expectancy compared with the general population. Previous research exploring SMI and diabetes highlights that people with SMI experience barriers to self-management, a key component of care in long-term conditions; however, this has not been investigated in the context of other LTCs. The aim of this study was to explore the lived experience of co-existing SMI and LTCs for service users, carers, and healthcare professionals.

**Methods:**

A qualitative study with people with SMI and LTCs, their carers, and healthcare professionals, using semi-structured interviews, focused observations, and focus groups across the UK. Forty-one interviews and five focus groups were conducted between December 2018 and April 2019. Transcripts were coded by two authors and analysed thematically.

**Results:**

Three themes were identified, 1) the precarious nature of living with SMI, 2) the circularity of life with SMI and LTCs, and 3) the constellation of support for self-management. People with co-existing SMI and LTCs often experience substantial difficulties with self-management of their health due to the competing demands of their psychiatric symptoms and treatment, social circumstances, and access to support. Multiple long-term conditions add to the burden of self-management. Social support, alongside person-centred professional care, is a key facilitator for managing health. An integrated approach to both mental and physical healthcare was suggested to meet service user and carer needs.

**Conclusion:**

The demands of living with SMI present a substantial barrier to self-management for multiple co-existing LTCs. It is important that people with SMI can access person-centred, tailored support for their LTCs that takes into consideration individual circumstances and priorities.

**Supplementary Information:**

The online version contains supplementary material available at 10.1186/s12888-022-04117-5.

## Introduction

Severe mental illnesses (SMI) are enduring mental illnesses, often with features of psychosis, and include schizophrenia, schizoaffective disorder, psychosis, and bipolar disorder. People with SMI experience higher rates of physical illness than the general population [1]. Their life expectancy is 15-20 years shorter [1–4] mainly due to coexisting physical long-term conditions (LTCs) [5–7]. Accessing clinically and cost-effective healthcare for individuals with a combination of mental and physical illness is recognised as challenging. Symptoms of SMI and LTCs and their treatments may interact to increase disease and treatment burden [8]. Antipsychotic medications are often used to treat psychosis in people with SMI and are associated with metabolic side effects. This can lead to significant weight gain, glucose dysregulation, hyperlipidaemia and as a result contribute to the development of metabolic syndrome [9]. Consequent health inequalities are exemplified by the experience of coexisting SMI and diabetes, where diabetes is two to three times more common [1, 10], and mental health and diabetes outcomes are poorer, than for individuals with diabetes alone [5–7].

Other common LTCs, including chronic obstructive pulmonary disease (COPD), ischaemic heart disease, and heart failure, all of which have a significantly higher prevalence among people with SMI [1]. While each LTC comes with its own set of symptoms and challenges, people with SMI often experience multiple LTCs [1]. Self-management of LTCs, the skills, practices, and behaviours that a person engages in to protect and promote their health, is fundamental to improving clinical outcomes [11–13]; and consistent self-management is essential for all LTCs to prevent worsening of symptoms and deteriorating health [14, 15].

Although reliable data are difficult to obtain, self-management support appears to be rarely offered to people with SMI and diabetes [16]. Moreover, the effectiveness of diabetes self-management programmes for this population is largely unknown as research typically excludes them [17–19]. SMI is characterised by disturbances of thought, perception, mood and motivation [20, 21], which influence self-efficacy, literacy, lifestyle, behaviour and family life [2, 22–26]. These disturbances can make self-management more difficult for people with SMI, and can be exacerbated further by more systemic issues such as stigma [27], diagnostic overshadowing, discrimination [28], housing insecurity, and poverty [29]. However, diabetes self-management programmes designed for the general population do not address these important barriers [30–32]. There is an urgent need to rectify this to avoid further widening of health inequalities [12].

The STEPWISE trial evaluated the clinical and cost effectiveness of a structured lifestyle education support programme to support weight loss in individuals with SMI [33]. Similar programmes have previously been shown to be effective in other populations. However, despite being tailored to the needs of people with SMI and being co-designed by people with SMI, the STEPWISE intervention appeared to meet the needs of study participants, it did not demonstrate a clinical benefit nor was it cost effective in supporting weight loss compared with treatment as usual. This study highlights the challenges of supporting people with schizophrenia to make sustainable lifestyle changes, with the view to improving their health long term.

The present study forms part of the DIAMONDS Research Programme [34] which aims to develop and test a tailored diabetes self-management intervention for people with SMI. The programme also sets out to develop a framework of transferable intervention components that may be generalisable to support people with SMI and other LTCs, such as COPD.

There remains an important gap in knowledge which relates specifically to a first-hand understanding of the lived experience of people with SMI and a range of LTCs, as well as carers, and the healthcare professionals involved in their care. This will offer important insights and implications for intervention design to support self-management of LTCs in this population. The present study reports findings from qualitative interviews with people with SMI and co-existing LTCs, carers, and healthcare professionals exploring this knowledge gap.

### Study aims

The aims of this study were to:

- identify and explore factors that promote or inhibit self-management behaviours in people with SMI and co-existing LTCs
- identify factors that may affect access to and uptake of self-management support and interventions
- explore use and acceptability of digital technologies for supporting self-management

## Material and methods

### Study Design

We undertook a qualitative study using face-to-face semi-structured interviews and focused observations with people with SMI and co-existing LTCs, alongside telephone semi-structured interviews and focus group discussions with informal carers and healthcare staff.

### Setting and recruitment

#### People with SMI and co-existing LTCs

People with SMI and co-existing LTCs were purposively sampled through NHS mental health trusts and primary care sites in the UK. The purposive sampling approach aimed to ensure representation of different SMIs and LTCs, with a range of severity. People were eligible for inclusion if they were over the age of 18, had a diagnosed SMI (defined as schizophrenia, schizoaffective disorder, bipolar disorder, or other non-organic psychosis; (corresponding with categories F20.0–20.9, F22.0–22.9, and F31.0–31.9 from the 10th revision of the International Classification of Diseases) [35, 36], and had at least one of the following diagnosed co-existing physical LTCs: cardiovascular disease (e.g. heart failure, ischaemic heart disease and cerebrovascular disease); metabolic disease (e.g. diabetes (except gestational diabetes), metabolic syndrome, hypothyroidism); respiratory conditions (e.g. asthma, COPD, emphysema); or chronic kidney disease. People were excluded from the study if they were in an acute psychiatric inpatient ward during the recruitment period, or if they lacked capacity to consent.

People with SMI and LTCs were recruited using three approaches. Firstly, eligible potential participants were approached directly during their consultations with healthcare professionals and staff working in general practices, mental health services, and third sector organisations. Secondly, patient lists of GP and mental health services were reviewed to identify potentially eligible people, who were then invited to take part. Finally, posters and flyers were placed in GP waiting rooms, mental health clinics, third sector organisation venues, and other appropriate locations. The study was also advertised on mental health trust and organisation websites and social media.

#### Informal carers of people with SMI and co-existing LTCs

Informal carers included, but were not limited to, partners, parents, other family members, or close friends, who may or may not live with the person that they support. Service users who agreed to participate in the study were asked to identify a relative or friend who supports them. Informal carers were also recruited from carer groups that were linked to participating primary care sites (or were identified by GPs or care coordinators), mental health trusts, and third sector organisations. Posters and flyers advertising the study were also aimed at informal carers.

#### Healthcare staff who provide care for people with SMI

Participating organisations were asked to identify staff who provide care to people with SMI and LTCs, and distribute information about the study via email and through posters in communal staff areas.

As part of the consent process, all participants gave permission for their interview/ focus group discussion to be audio recorded.

### Data collection

We conducted one-to-one, face-to-face (in-person) semi-structured interviews with 32 people with SMI and LTCs (“service users”), with a mean age of 54.4 years, ranging from 24 to 76. In addition, we held two focus group discussions with informal carers (four and three participants, respectively) as well as five one-to-one semi-structured telephone interviews. Healthcare professional views were gathered in three focus group discussions (two with four participants from mental health trusts and one with six participants from a range of physical health services and primary care) and four one-to-one semi-structured interviews. All interviews were conducted by one of three researchers with qualitative research experience. The same researchers also facilitated the focus group discussions, working alone or as a pair. All semi-structured interviews and focus group discussions were conducted between December 2018 and April 2019.

Interviews were based on topic guides providing an outline of questions to be asked as well as suggested prompts to allow participants to elaborate on pertinent aspects of their experience (Appendix A). The topic guides were developed to ensure we explored a broad range of influences on self-management behaviours. In addition, a visual storyboard was used in the service user interviews to elicit further, more detailed responses about experiences of self-management (Appendix B). The topic guides and storyboard were developed and refined in collaboration with the patient and public involvement group DIAMONDS Voice [34].

Focused observations involved a two to three-hour session where the researcher spent time with the participant doing an activity of their choice that may have had relevance to self-management, for example food shopping, cooking, walking in the park, attending a community group, attending an appointment with a health professional, or reading health information on the internet. During the session the researcher both observed and asked questions about the activity that could not be answered by observation alone. Immediately following the session, the researcher took focused field notes about the activity and how this related to SMI and LTC self-management behaviours and the factors that affected these. Focused observations took place between December 2018 and April 2019.

### Qualitative Analysis

#### Data management

Audio recordings of the interviews and focus group discussions were transcribed verbatim by a third-party transcription service. Transcripts were imported to NVivo12 [37] and anonymised prior to coding and analysis.

Service user transcripts and field notes were grouped into cases based on the most salient LTC diagnosis discussed in the interview: metabolic condition (n=12), respiratory condition (n=13), cardiovascular condition (n=3), and “other condition” (n=4). Carer and healthcare professional transcripts were not divided into cases and instead treated as distinct groups of transcripts in their own right.

All service user participants who had completed a semi-structured interview were offered the opportunity to participate in focused observation. Seven service users agreed to take part, of whom four had a metabolic condition, one a respiratory condition, and the remaining two were included in the “other” category. Field notes for the structured observations were imported to NVivo12 [37].

#### Coding

Service user transcripts were coded by two out of three researchers with the necessary experience in the field, including an experienced qualitative researcher with a background in mental health nursing, a PhD student with extensive qualitative experience and in-depth knowledge of the subject matter, and a novice qualitative researcher. Transcripts relating to carer interviews/ focus group discussions were coded by one researcher, those relating to healthcare professionals by another. All coding and analysis were supervised by a senior qualitative researcher.

Initially, six service user transcripts were coded inductively with codes generated from the content of the transcripts. To streamline the data management processes, these codes were mapped onto an existing coding framework that had been used for a contextually related study by our team [38]. The coding framework was refined in several iterations through discussions within the team. Irrelevant codes were removed and new codes, particularly related to the use of digital technology, were added in order to address the objectives of the research. The final version of the coding framework can be found in Appendix C. Similarly, coding of the carer and healthcare professional transcripts was based on a coding framework derived from related previous work with codes added or removed in team discussions as necessary. The observational field notes were coded by one researcher within the same established coding framework.

#### Thematic analysis

While service user transcripts and field notes were initially coded within their allocated LTC case groups, reading codes across the cases revealed that the lived experience described by service users did not differ in ways directly related to the nature of their LTC diagnosis. As such, we did not continue with a separate analysis for each case and instead proceeded with a thematic analysis of all service user data. Thematic analyses of carer and healthcare professional transcripts were conducted separately [39].

Once the initial coding was completed within the organising structure of the coding framework, we combined the transcripts from all groups and inductively generated themes and subthemes from the list of codes. This process was led by one researcher, with proposed themes and subthemes reviewed and revised with the rest of the team in an iterative process. Codes from the observational field notes fed into the generation of themes alongside the interview transcripts.

## Results

### Themes and subthemes

Our analysis revealed high levels of convergence across interview and focus group data from service users, carers, and healthcare professionals, pointing to three core themes that centred on the challenges (and enablers) of supporting people with SMI to self-manage their physical and mental health. The themes along with associated sub-themes are described in Table 1.

**Table 1:** Overview of themes and subthemes

Theme	Description of themes	Subthemes
1. The precarious nature of living with SMI	Focused on how the constant unpredictability and overwhelming nature of mental illness lessens opportunities for self-management of LTCs.	SMI is inescapable SMI is a ‘rollercoaster’ disease Short-term needs are prioritised over long-term self-management
2. The circularity of life with SMI and LTCs	Articulated how the efforts to manage physical LTCs often reinforced the centrality of mental health	Sacrificing physical health to manage mental health LTCs add to an already substantial burden Physical and mental illness are enmeshed
3. The constellation of support for self-management	Centred on understanding the multiplicity of care structures that might help people with SMI to manage their physical and mental health	Sources of support Supporting the whole person Unmet support needs

### Theme 1: The precarious nature of living with SMI

#### SMI is inescapable

People with SMI commonly expressed how the relationship with their mental illness was ever present, often fraught, and far reaching, impacting all aspects of their lives. With no end or relief in sight, service user participants described how the presence of SMI was inescapable, *“It’s upsetting knowing that it’s never gonna go away” (Service user, PLGP012),* representing a relentless and never-ending struggle:

*Well, it’s all the time, I mean, when you’ve gone from here, it’s still gonna be there all today, again tomorrow, next week, this week. Never goes. - Service user, PNSCH003*

Beyond the enduring nature of SMI, the onset of psychotic symptoms such as delusions or hallucinations signalled highly distressing breaks with reality that exacerbated the sense that the impact of SMI is unrelenting:

*Sometimes she hears voices, and this is the, this is what causes the problem, the voices really bring her down, and they’ve not got to the bottom of the voices - Carer, CKMPT002*

Healthcare professionals also described the negative impact of hallucinatory symptoms on service users’ capacity to manage their mental health. In these contexts, support for managing physical health is markedly absent:

*…it was a patient with Schizophrenia who had real problems with partly cultural beliefs but also auditory hallucinations telling them not to take the medication, it’s poison and it doesn’t help and that the Nurse that comes is trying to poison you. - Healthcare professional focus Group 1*

The constant threat and challenges of mental health symptoms also intruded on the lives of carers who voiced that they were similarly preoccupied with the sense that SMI had an inescapable hold on their lives too:

*… they used to say to me a few years ago “...she could go in a flat on her own … if it gave you a break ….” And I went, … “She’s got severe mental health issues, … she’s bipolar, what if she’s in a really bad mood one day and she… does something… - Carer focus group 1*

#### SMI is ‘a rollercoaster disease’

While many service users described periods of comparatively good mental health and reported being well when interviewed, there was a strong current in the data highlighting that SMI was “*…a rollercoaster disease…*” (Service user, PLYPT004), with unpredictable periods of exacerbation and amelioration:

*I can become quite ill at a moment’s notice, with my head, I can be climbing the wall sometimes and I mean it, climbing the walls and furniture and not knowing where I am, what I’m doing. -Service user PLYPT004*

Reminiscent of Charmaz (1991) [40], service users talked of good days and bad days, and how they routinely experienced fluctuating fortunes that stemmed from the episodic nature of their symptoms. The persistent precarity of living with SMI was also felt by carers who recognised that good days could be swiftly followed by bouts of ill health:

*Every now and then, you’ll have a good period. Over Christmas, she had a good period where because it was Christmas, we were meeting all the family….. But then as soon as Christmas is over, Boxing Day, she crashed.[…] And she was bad then for probably two weeks. - Carer focus group 2*

The uncertainty and burden associated with SMI symptoms meant service users were sometimes drawn to maladaptive coping strategies, often to the detriment of their physical health.

*I drink every day. And depending how my mental health is especially, it depends on you know, how much I’m drinking. So, when things are better, I sort of have like a measured amount. Whereas, when things are not good, it’s just escapism really - Service user, PLGP007*

Healthcare professionals recognised that physical health was often of secondary importance for service users whose desire to manage distressing mental health symptoms led them to engage in health risk behaviours such as smoking and drinking. Carers also witnessed instances where mental health took precedence over physical health, with many acknowledging that harmful coping strategies were normalised in the face of acute episodes:

*If … she doesn’t smoke she’d be even worse…. we had a situation …when she was in the doctors she had one of these attacks and she was in a state at the doctors, and even the doctor came and lit a cigarette for her which calmed her down. So, you know, … smoking is actually…medicinal rather, rather than anything else. – Carer, CKMPT002*

In this context, professionals tasked with supporting physical health were often unable to recalibrate the focus of service users whose preoccupation with mental health was at the exclusion of physical health:

*… a lot of people I see don’t think their physical health’s really important, they’re more concerned about mental health, so when I ring them and say, we’re going to come out and do a physical health assessment they go well why do you need to do that? What’s so important about that? - Healthcare professional focus group 3*

#### Short-term needs are prioritised over long-term self-management

A contributing factor to service users’ inability to recalibrate their focus was the immediacy of mental health symptoms underpinning the prioritisation of short-term needs and goals over long-term self-management of physical health. Indeed, rather than self-management, service users often talked about the struggle to engage in practices that were essentially about basic self-care. Undertaking activities of daily living was a goal in itself, often leaving no capacity or capability to apply themselves to more demanding tasks such as meal planning or cooking healthy food:

*That’s the only way that I can cope from day to day, I try to do what I can, but some days it’s too hard to do even the basics. I don’t do any cooking for myself, or anything like that, that’s all done for me, or I live on things that are straight from the fridge. -Service user, PLGP014*

Likewise, carers recalled many instances where their focus was essentially on supporting service users to navigate basic tasks such as dressing and feeding, especially in the face of debilitating mental health symptoms:

*Some days it can prevent him from getting out of bed … and then it can be a challenge just to get [son] up and to eat something …. I have known days where a culmination of his anxiety and hallucinations, it can be a battle just to get him to have a drink and a slice of toast. -Carer, CLCFT010*

These descriptions demonstrate that the experience of living with SMI feels precarious. Service users are living with inescapable, overwhelming symptoms that fluctuate in severity on a regular basis. As a consequence, engaging in risk behaviours such as drinking alcohol or smoking is viewed as a valid coping mechanism as it reduces the distress associated with SMI symptoms. The precarity of SMI also extends to the difficulty many people have meeting their basic needs, meaning that their immediate need to eat, drink, and wash is prioritised over self-management behaviours for their long-term physical health.

### Theme 2: The circularity of life with SMI and LTCs

#### Sacrificing physical health to manage mental health

The centrality of mental health was further illuminated by service users’ discourse about the interconnected and compound relationship between their SMI and their LTCs. Many service users shared a perception that the long-term management of their mental health problems had damaged their physical health:

*Kidney disease is something that’s ongoing because of my Lithium because I’ve taken Lithium for … 20 odd years. And that causes your liver, your kidneys to sort of not produce. –Service user, PLYPT008*

These reflections on the relationship between managing mental health and the onset of physical health problems extended to an appreciation that antipsychotic medication could lead to distressing side effects, not least weight gain and obesity:

*I am on Aripiprazole for that …, which I don't like at all because it’s given me severe weight increment issues… So I’m actually wanting to stop taking the Aripiprazole, I’m on a low dose but I still feel it’s high enough to cause me weight issues. –Service user, PLYPT007*

Carers often struggled to balance the health benefits of antipsychotic medication with the negative consequences of such medication, leaving them in a quandary about how best to support service users:

*the tablets don’t help, the tablets … make her put weight on so what do you do, you know? You’re in a Catch-22; if I stop that this happens, if I do that, you know.. - Carer, CKMPT002*

By contrast, service users, especially those who have had traumatic and negative experiences of specialist mental health services, invoked a more pragmatic approach to taking antipsychotic medication. Despite the risk of side effects such as weight gain, they rationalised taking antipsychotic medication on the grounds that it reduced the risk of involuntary hospital admission:

*I feel when I take the medication is, I don't want to … ever go back into psychiatric hospital, so I take it’s a condition to keep me in the community and I… don't ever want to go back into that. – Service user, PLPFT001*

#### LTCs add to an already substantial burden

Whilst service users primarily defined their lived experience through their mental health symptoms, they also recounted how the presence of LTCs, and especially multiple LTCs, profoundly affected their quality of life. Pain, fatigue, and disability conferred additional burden on service users and their carers. For some service users, pain precluded them from being physically active:

*I can’t exercise because of my legs, I can’t manage it. I can’t bend over properly, if I am on the floor I have to use something to get up. I am in pain with this leg all the time -Service user, PLGP002*

Additionally, several service users were deeply fatigued by symptoms associated with LTCs, preventing them from attending to personal hygiene:

*It’s not that I don't like having a shower but sometimes … I can put it off because I just get… over-tired, it just doesn’t seem to be a big necessity even though I know it is. -Service user, PLYPT007*

Moreover, in addition to the health impacts on service users, disability that stemmed from LTCs materially affected the quality of personal relationships, as indicated by carers whose domestic arrangements led them to sleep apart from their partner:

*…the mobility’s really hard; it’s hard to see, it’s hard for me because we, we don’t share a room anymore, he’s in his own room… he’s got his specialist bed and I’ve got my own room; … I must admit it feels a little bit sad, and I do struggle with it a lot. – Carer focus group 1*

Carers’ capacity to cope with these additional burdens is also often stretched even further by the need to support service users to manage the complex array of healthcare for LTCs:

*The only other problem is juggling all his appointments for the neurologist, for the epilepsy and …. – Carer focus group 2*

#### Physical and mental illness are enmeshed

Whilst these accounts point to the significant impact LTCs have on the physical health and quality of life of service users and carers, the overriding narrative about managing LTCs was underscored by reflections about impacts on mental health. In this sense, perspectives about the relationship between SMI and LTCs came full circle and further emphasised the centrality of mental health to the lived experience of service users and carers. This was evidenced where carers recalled how service users might feel a heightened sense of stress when tending to physical health problems, potentially leading to worsening mental health:

*He’s got liver cirrhosis and also, due to his poorly liver, it triggers off a lot of mental health issues. So if he was in a stressful scenario or if he’s in public or anything …he’s had episodes of becoming quite psychotic - Carer focus group 1*

Service users also recalled moments when their LTC was not well managed or even diagnosed and they encountered frightening physical symptoms that could lead to anxiety and panic:

*And I hadn’t been diagnosed with that COPD then. So, by the time I got to the top of them stairs, I were that out of breath I were nearly going to a panic attack. -Service user, PLGP008*

Despite awareness that LTCs could affect their mental health, service users were at times resistant to taking medication to manage their physical health problems. Compared with the medical management of SMI, which necessitated long-term use of antipsychotics, some service users reported only taking medicines for their LTC on an irregular basis.

*So, the only time that I end up really taking stuff is if I’ve got a bad cold or a chest infection where you know, I really do sort of need it to breathe. - Service user, PLGP007*

Other service users, in the context of managing their SMI through medication, expressed a desire to remain as free of medical treatment as possible. This was true even where side effects from medication such as statins were minimal and the benefits for reducing risk of cardiovascular disease could be high:

*I don’t need it, I think nature will keep my cholesterol low. I believe that… you can’t beat nature, it’s the perfect designer. The fewer pills and drugs I take the better it is. - Service user, PNWBH001*

The desire to avoid medical treatment for physical health conditions extended to avoidance of healthcare services as a whole. Prior traumatic experiences as a result of paternalistic mental health care, including detention under the Mental Health Act 1983 [41], resulted in some service users avoiding healthcare professionals in general.

*I’ve been really honest with people about stuff and I’ve been treated badly in hospital for it. And in a day hospital and in the acute hospital as well so that puts me off seeking help sometimes because of the way I was treated really. When I’ve done nothing wrong. I were ill and they treated me like a criminal. -Service user, PLYPT003*

Other service users recounted difficult experiences where they faced significant stigma from within healthcare services, or where their mental health issues were dismissed or minimised.

*I did sort of say to one of the midwives and she was like, “Oh no, it’s just baby blues.” And then I ended up sectioned in mother and baby unit. Yeah. It wasn’t good. -Service user, PLGP007*

These experiences coloured their perceptions of healthcare professionals, reducing their willingness to reach out for any form of help, including for physical health, for fear of the treatment they would receive,

*These aren’t nice places [psychiatric wards]… often not nice because of the staff and that’s really heart-breaking to think and to come forward again, because it’s mental health and all the stuff attached to it. - PLGP004*

The experiences of SMI and LTCs are difficult to differentiate as they feed into each other, reinforcing the challenges and creating a cumulative burden. Service users, carers, and healthcare professionals face decisions around psychiatric medication with significant physical health implications, increasing the risk of developing LTCs. In turn, LTCs create an additional symptom burden that compounds existing challenges of self-management. Simultaneously, people with SMI experience traumatising treatment from mental health services, reducing their willingness to engage in any form of healthcare. Ultimately, this dynamic can lead to a deterioration in both mental and physical health.

### Theme 3: The constellation of support for self-management

When considering factors that might enable or inhibit self-management of physical and mental health among people with SMI, our analysis initially addressed a set of seemingly disparate factors related to social, instrumental, and structural forms of support. However, a recurring theme within these data, especially from carers and health professionals, was how holistic support models functioned or could function to not only support service users to engage with self-management but to also support carers. In this sense, these data spoke about a constellation of supportive mechanisms and components that underpinned service users’, carers’, and healthcare professionals’ perspectives about the work of self-managing LTCs in the context of SMI.

#### Sources of support

Discussions about self-management of LTCs in SMI were played out against a broader understanding on the part of healthcare professionals that the combination of mental and physical health problems “*…is very difficult”* and managing *“those problems together would be a tall order [for anybody]*” (Healthcare professional, SLPG004). Critical to this task is attending appointments and healthcare professionals recognised that accessibility of their services was difficult for people with SMI, with knock-on consequences for their physical health:

*Sometimes they can’t engage with these appointments if they have got a chaotic lifestyle, so they might miss appointments and then there is no sort of follow up for that so they miss out on appointments or investigations. - Healthcare professional, SLCFT001*

Additionally, quite apart from issues about engagement with services, carers expressed concern that people with SMI and physical health problems were not experiencing equitable access to healthcare, leading to damaging consequences for their health:

*...she had a twisted spinal cord, they had to operate, and … when I wheeled her into the doctor, the surgeon, had to do it in a wheelchair because she couldn’t walk and the doc, the surgeon said “… how did you get in this state?” She said “Because nobody believes me.” But it’s not just confined to her, it’s a regular thing for other people that we know that have got a mental illness, they’re not treated the same.-Carer, CKMPT002*

Healthcare professionals also recognised that to remedy lack of engagement with appointments, people with SMI needed the right forms of support to address physical health problems adequately:

*…but on the whole it’s [physical health] very poorly managed mainly because they don’t engage in primary care services, whether it be that they need support in going to attend their appointments or they have a lack of understanding about their physical health and wellbeing and they need extra support and education and advice about this. - Healthcare professional, SLCFT001*

Informal support networks of family and friends were seen by healthcare professionals as key to supporting people with SMI to attend appointments for their physical health.

*I do think the support system thing is vital, I know of several patients who have partners and family who do their dandiest to encourage regular attendance and to do it for them to a large extent. - Healthcare professional, SLGP001*

Furthermore, carers viewed their ability to not only help service users attend appointments but also to understand and remember health information as essential to good management of physical health:

*Well, I take [name] to appointments and I go in with him. I go in because by the time he’s got to the main door after he’s seen a doctor, he’s forgotten what they’ve said to him, more often than not. … So, I’m there to find out, okay, what is this? What’s going on? What’s happening here? What are you gonna do about this? And then we get outside, we’ll sit in the car and discuss it. - Carer focus group 2*

Similarly, for service users, unused to interpreting health information or unsure about optimal LTC self-management, being able to draw on the support of friends was a critical component of their support network:

*My friend is diabetic, she lives over the road from me and I’m always turning to her and asking her, I just did my blood sugar, is this right, is this wrong? So she advises me, she’s had diabetes for more than ten years, so I have a little support system with her – Service user, PLGP005*

#### Supporting the whole person

Considering the needs of the person as a whole [42], as opposed to compartmentalising different aspects of health, social and economic circumstances, was seen as necessary to address health inequalities. At a service level, there were many instances where healthcare professionals pointed to the need to reconfigure the way secondary specialist health clinics operated, to free up more time to address the complex and compound impact of SMI and LTCs.

*You know our service which sees people once or twice after a cardiac event was never ever going to be, you know sufficient for this lady. You probably need to take months and months of seeing her every week to get any kind of stability for her. – Healthcare professional focus group 3*

Similarly, in primary care, healthcare professionals were cognisant of gaps in service provision to ensure that both physical and mental health of service users was addressed, especially in the absence of informal support or dedicated support workers*.* Service users endorsed approaches that were more integrated with many recounting that they benefited from seeing healthcare professionals who were able to not only deal with physical healthcare, but were also able to offer time and connect with mental health services if necessary:

*I have a community nurse that comes every fortnight who gives me my injection and she will sit with me for a good half an hour and I can offload to her and it’s really good that I can have that time and if anything goes wrong, she can get me an appointment to see my psychiatrist, straightaway if I need to. – Service user, PNECO003*

With the benefit of time and a more patient-centred approach comes opportunities to support people with SMI to participate in healthy activities that mapped to their needs and preferences:

*“But I make sure that it’s stuff that they want to do. … I’ll say what would you like to do to make yourself better? Some will pick swimming, some will just be going out for a walk... So, they’re not coming out with just stuff they’re going and doing something for themselves.” – Healthcare professional focus group 3*

However, healthcare professionals who were familiar with the constraints and contexts within which people with SMI lived often had to adapt physical health advice to be feasible and realisable:

*“… if somebody is living on the street then I can’t really advise them to go to the gym, so we have to like-- we have to be pragmatic about what advice we give with our patients.” – Healthcare professional, SLGP004*

This informed and tailored approach to supporting self-management of LTCs contrasts markedly with the experience of some service users who only received detached, non-specific, and one-off advice:

*I’ve got a diabetic nurse at my surgery, she basically gave me some leaflets and I didn’t have that much information on when I should be eating, what I should be eating and she just sort of gave the leaflets and it was off you go, kind of thing, -Service user, PLGP005*

#### Unmet support needs

In an echo of the previous theme about the circularity of lived experience, there was also significant resistance from service users about the use of digital services to communicate advice about physical health, not least because the use of web-based communication might prompt psychotic symptoms:

*I wouldn’t want none of that internet, people sending me emails, making me paranoid. I wouldn’t want nowt like that. - Service user, PLGP008*

Digital exclusion was also a key consideration for why healthcare professionals had struggled to generate engagement and interest among people with SMI for digital applications:

*We got that, My COPD app that we’re trying to roll out. We’re finding it quite hard to find enough patients that have an email address, that know how to use a computer, that can use a smart phone because the population of the type of patients either can’t afford it, don't have it, not interested in it or you sign them up and then never use it. - Healthcare professional focus group 1*

Whilst there was an emerging consensus about the levels of formal and informal support people with SMI might need to optimise self-management of LTCs, there was less certainty about how to support carers’ health and wellbeing:

*Hospitals and professionals, they need to think about carers and have a word with them and find out what their needs are, take their contact details, and realise that they will have needs as well, cos when you support these kinda people then you don’t want it affecting your life and your health and stuff like that, but it does have an effect on your life and your health too.– Carer, CRDASH002*

The lack of support for carers was keenly felt among older adults who cared for their adult children with SMI:

*But the problem is we’re not getting any younger … if anything happens to us she won’t be here. And I mean that…It is a worry cos I’m nearly, I’m seventy-eight now, seventy-seven, and it is a worry.*

*Q: Yeah. She depends on you very much?*

*A: Too much, too much; - Carer, CKMPT002*

These reflections from carers further illustrate that to optimise the self-management of both mental and physical health among people with SMI a constellation of support is required that extends across primary, secondary and community health services, including informal care and social support, as well as support from and for carers.

## Discussion

This qualitative study explored the lived experience of people with SMI about the challenges of living with co-existing LTCs and mental ill health, and sought to understand how carers and healthcare professionals accommodated these challenges with a view to supporting self-management of LTCs. Our findings showed that the inescapable presence of SMI symptoms dominated the experience of service users, whose lives were characterised by unrelenting precarity – the only apparent certainty was the sense that their mental health was always in the balance, oscillating from crisis to periods of relative stability. The episodic and sometimes traumatic nature of living with SMI precipitated a focus on short-term goals only, often to the exclusion of managing LTCs. Even where LTCs were acknowledged, we found that service users often attributed their physical health problems to the treatment of their SMI. The carer voice addressed how LTCs imposed additional burden on their lives and relationships with service users. However, carers and service users also acknowledged that considerations about managing physical health were interlaced with fears about deteriorating mental health, bringing the narrative about lived experience full circle to symptoms of SMI. In this context the perspective of healthcare professionals pointed to the need for holistic forms of support to prompt and sustain self-management of mental and physical health in people with SMI. This constellation of support was shown to function at a formal and structural level, but also drew on informal and social forms of support, showcasing the need for more joined-up and comprehensive care for people with SMI and LTCs.

Our findings resonate with a recent qualitative evidence synthesis that explored facilitators and barriers to self-management of physical health among people with SMI [43]. This review synthesised findings from 21 studies and highlighted that SMI symptoms placed a high burden on service users who struggled with side effects of antipsychotic medication and found it difficult to get out of the house. Other comparable findings from this synthesis illustrated how the management of physical health could negatively interact with mental health. As with our study, the review found that critical to breaking this cycle of being dominated by SMI symptoms was the input of healthcare professionals with time and social and instrumental forms of support. However, the review only included six studies of people with SMI and LTCs, all of whom had diabetes or cardio-metabolic conditions. Similarly, previous qualitative work has highlighted that self-management relied on the provision of a large network/constellation of support for everyday challenges experienced by people with SMI and diabetes and their carers [38]. Our study extends these findings and shows that the challenges of self-management among people with SMI translate across all LTCs, pointing to the need for generalisable solutions.

Furthermore, our findings about the importance of a broader supportive environment fits with the results from a systematic review that mapped the evidence for psychological and social determinants of self-management behaviours in people with SMI [44]. Evidence from this review showed that environmental context and resources were a critical determinant of self-management behaviours associated with good physical and mental health (e.g. medication taking, being active, reducing risks, healthy eating). However, this review included only four studies of people with SMI and LTCs, which were exclusively about the additional impact of diabetes. We have shown here that environmental constraints and facilitators are relevant to supporting self-management in people with SMIs and multiple LTCs.

A striking finding across our data (and previous qualitative studies about lived experience of SMI) [38] was the primacy of day-to-day struggles with activities of daily living, leading to a focus on short-term time horizons at the expense of long-term management of physical and mental health. Similar themes have been noted in qualitative syntheses of the lived experience of multiple long term physical health problems [45]. However, unlike in the context of physical multimorbidity, where people with multiple LTCs are able to tactically deploy know-how to manage symptoms, we found that service users were constrained by their short-term battles with their symptoms and with themselves. Moreover, the tension between getting through each day and strategically managing health over the long term is magnified for people with SMI because prioritising treatment of mental health with antipsychotics can lead to long-term damage of physical health.

A focus on short-term goals is consistent with a “present time” perspective. Grounded in socioemotional selectivity theory, time perspective is proposed to play a fundamental role in goal formation and psychological functioning, and has important implications for self-regulation of emotions and coping over the life course [46]. Unlike future time perspectives, which are oriented towards planning for the achievement of a future goal, a negative past and fatalistic present time perspectives have been shown to be more positively correlated with depressive and anxious moods [47]. In the presence of crisis and insecurity, present time perspectives have been shown to be the optimal adaptive and coping strategies [48]. We saw such fatalistic present time perspectives in service users’ accounts about focusing on self-care tasks and in coping with mental health by adopting risky behaviours such as drinking and smoking.

Fear of experiencing further highly distressing and frightening encounters with healthcare professionals and services also in part accounted for a focus on short-term needs and a lack of engagement in health care services and self-management. Drawing on the sociology of chronic illness, Gately et al. [49] have shown that how people with LTCs engage with health services is mediated by the patient’s life world, often characterised by the complex and recursive nature of encounters with healthcare. This is especially true in relation to people with SMI and LTCs whose previous encounters with mental health services might have involved paternalistic practices such as restraint and other stigmatising behaviours [50], leading them to avoid engagement with healthcare [51, 52], even for debilitating LTCs.

### Implications for research and practice

Managing co-existing mental and physical conditions presents a range of challenges for patients, informal carers, and professionals. Our data demonstrate the need for more holistic care models that can support self-management, not least through the provision of services that afford additional time to accommodate competing priorities. Most people with SMI are managed in primary care [53] and it has been shown to be possible to reconfigure the service in primary care to allow for longer consultations, relationship continuity, and self-management support when managing people with multimorbidity from deprived areas [54]. There is scope to enhance service user experience of primary care for management of multiple co-existing conditions through similar service-led innovations. Additionally, in the absence of social support and to relieve burden on carers, there is call for further work on the benefits of using peer navigation approaches to support integration of mental and physical health in people with SMI. This approach has proven utility to reduce health disparities in ethnic minorities with SMI [55] and could support people with SMI and LTCs to engage with and negotiate complex health information across primary and specialist care settings.

Understanding the social and psychological determinants of health and risk behaviours among people with SMI and LTCs can also point to the potential mechanisms of action and behaviour change techniques that might support self-management [56]. There is an opportunity to build on these findings to identify and test behaviour change techniques implicated in, for example, a person’s situation or environment that discourages or encourages self-management behaviour.

Further research is also needed to better understand traumatising negative experiences within mental healthcare and the role these may play in health inequalities for people with SMI. Trauma-informed care within mental and physical health services, efforts to eliminate pervasive stigma amongst healthcare professionals, and adhering to principles of least-restrictive practice [57], may help reduce the risk of iatrogenic trauma and promote engagement with services. This also ties in with the need to build in service user and carer involvement in physical health care discussions as part of mental health care planning in a strategic and structured way [58]. How best this is achieved is still uncertain but new models of primary and integrated care that adopt a whole person approach offer service level solutions that might support people with SMI to better manage the physical health challenges [59]. These care models need robust experimental evaluation and refinement based on qualitative process evaluation.

### Strengths and Limitations

These findings address a gap in the existing literature, as the majority of the available evidence on the experience of co-existing SMI and LTCs centres on type 2 diabetes. The cohort of service users, carers and healthcare professionals that were recruited into this study had experience with multiple LTCs, including metabolic, respiratory, and cardiovascular conditions. Our study thus extends the evidence base beyond diabetes and illustrates that people with SMI and multiple LTCs struggle to live beyond the impact of their mental illness. They need additional forms of formal and informal support to encourage uptake of self-management. This study also benefited from source triangulation. By collecting data not only from service users, but also from carers and healthcare professionals drawn from a range of specialties, we were able to provide a more comprehensive and integrated understanding of encounters with SMI and LTCs.

We did not collect comprehensive demographic data from service users and carers, which limits our capacity to tease out relationships between data and individual/socioeconomic characteristics. Most carers who participated in this research were not the care providers for service user participants, and therefore carer accounts were often detached from service user data. However, this did allow us to capture experiences of SMI and co-existing LTCs where the service user would not have been well enough to participate in the research themselves. Additionally, because carers were often unrelated to service users, it was difficult to identify and recruit this group, reflecting a broader issue of the involvement of carers of people with SMI in research. Future research could address this issue by approaching carer groups, instead of relying on mental health and statutory services as sources of recruitment.

## Conclusion

Living with SMI and multiple LTCs is defined by unrelenting precarity that confounds efforts to engage with health care services and self-management, an issue that is consistent regardless of the type of LTC. We showed that people with SMI and a broad range of LTCs are often entrapped in a lifeworld characterised by a sense of circularity - no matter the starting point, their perceptions and experiences tend to return to and revisit life with SMI. To break through these limitations, we highlighted that people with SMI and LTCs need access to a constellation of support that includes structural, instrumental, and social forms resources, contacts, and networks. Future research should focus on interventions that can address the need for person-centred, comprehensive support for service users, as well as improving and increasing the support available to informal carers. The DIAMONDS Programme endeavours to take a first step in this direction by designing and testing a tailored diabetes self-management intervention for people with SMI.

## Supplementary Information

**Additional file 1.**

**Additional file 2.**

**Additional file 3.**

## Data Availability

The datasets used and/or analysed during the current study are available from the corresponding author on reasonable request.

## Abbreviations

COPD: Chronic obstructive pulmonary disease
GP: General practitioner
LTC: Long-term condition
NHS: National Health Service
SMI: Severe mental illness
