# Supplementary material for: The lived experience of severe mental illness and long-term conditions: a qualitative exploration of service user, carer, and healthcare professional perspectives on self-managing co-existing mental and physical conditions
Source: BMC Psychiatry. 2022 Jul 19;22:479. doi: 10.1186/s12888-022-04117-5 (PMC9295434; doi:10.1186/s12888-022-04117-5)
Supplement: Supplementary file 1 — Additional file 1. [file 12888_2022_4117_MOESM1_ESM.docx]

**Appendix A. Topic Guides**

**Outline Topic Guide: People living with SMI and LTC interviews**

1. **Background information**

Current health conditions, including when they were diagnosed

Current health status (e.g. how their conditions are, how their health is)

Current medications / care / interventions received for LTC and SMI

1. **Managing LTC alongside SMI**

Explore the advice and information participants have received about their own role in managing their health, and the sorts of activities / behaviours they think they are supposed to do

Explore these activities and behaviours with participants over a recent two-week period, including reasons why they have and haven’t done them

Use the visual storyboard to explore their behaviours, knowledge, beliefs and perceived consequences for the AADE seven self-care behaviours (diet, exercise, monitoring, treatment adherence, problem solving, risk reduction and healthy coping, which are visualised for participants with images selected to act as prompts and points for discussion)

Barriers to LTC self-management (use TDF domains as prompts)

Enablers for LTC self-management (use TDF domains as prompts)

Explore how SMI symptoms impact on managing LTC and vice versa

1. **Support for self-management**

*How each of the sources of support listed below support or hinder self-management and what the participant would like in terms of these types of support:*

- Involvement and role of relatives/ friends in supporting self-management
- Involvement and role of healthcare professionals in supporting self-management
- Role of other services / support groups / community initiatives in supporting self-management
- Role of digital technologies (e.g. health apps, internet sites, telehealth)

**4. Demographics**

Demographic information: age, gender, employment, ethnicity, education experience, family and home circumstances, perception of neighbourhood, relative financial situation, social activities

**Outline Topic Guide: Healthcare staff**

| **Timing** | **Topic and Content** |
| --- | --- |
| 0-5mins | **INTRODUCTION POINTS**   - Introduce the facilitators / interviewer - Introduce the research and thank them for taking part - Talk about the purpose of the focus group/interview and how the information will be used - Set ground rules for focus groups - Introduce discussion topics   **Any questions?** |
| 5-35mins | **Role and experience in supporting self-management of LTCs in people with SMI**   1. Participants to introduce themselves for the purpose of the recording: name, job role, summary of experience / role in supporting LTC management in SMI 2. What does ‘self-management’ mean to you and what sorts of patient behaviours and activities does it involve? 3. Tell us about the advice and support you and the service you work for provide patients with SMI and co-existing LTCs about managing their health at home and how helpful you think this is |
| 35-65mins | **Determinants of self-management of LTCs**   1. How well do you think people with SMI manage their LTCs and are there certain self-management activities / behaviours that are more or less challenging for them? 2. How does SMI itself (e.g. symptoms, treatments) impact on self-managing a LTC? 3. What factors prevent, or help (e.g. barriers, enablers) these patients to manage their LTC?   *Prompts: capabilities (e.g. knowledge, skills, memory, cognition, behavioural regulation); opportunities (social influences, environmental context and resources); motivations (beliefs about capabilities and consequences, identity, optimism, goals, intentions, emotion, reinforcement)* |
| 65-80mins | **Role of digital technologies**   1. Discuss the current and potential role of digital technologies (e.g. mobile apps, telehealth and web-based applications) to support self-management of LTCs for people with SMI   *Prompts: acceptability, barriers, email and text communication, monitoring symptoms / activity* |
| 80-90mins | **CLOSE SESSION**   - Summarise key points - Remind participants about confidentiality - Thank participants |

**Outline Topic Guide: Relatives and friends**

| **Timing** | **Topic and Content** |
| --- | --- |
| 0-5mins | **INTRODUCTION POINTS**   - Introduce the facilitators / interviewer - Introduce the research and thank them for taking part - Talk about the purpose of the focus group/interview and how the information will be used - Set ground rules for focus groups - Introduce discussion topics   **Any questions?** |
| 5-40mins | **Role and experience in supporting self-management of LTCs in people with SMI**   1. Participants to introduce themselves for the purpose of the recording: name, diagnoses of the person they support and their relationship to them (e.g. spouse, friend) 2. Tell us about the activities (e.g. taking medications, monitoring symptoms, leading a healthier lifestyle, making appointments, attending groups) that the person you support does on a regular basis or is supposed to do for their LTC 3. Tell us about the advice, support and help you provide your relative/friend with SMI and co-existing LTC to do these things 4. How are you currently supported in this role and how would you like to be supported? |
| 40-65mins | **Determinants of self-management of LTCs**   1. How well does the person you support manage their LTC and are there certain self-management activities / behaviours we have discussed that are more or less challenging for them? 2. How does SMI itself (e.g. symptoms, treatments) impact on having and managing a LTC? 3. What do you think prevents, or helps the person you support to manage their LTC?   *Prompts: capabilities (e.g. knowledge, skills, memory, cognition, behavioural regulation); opportunities (social influences, environmental context and resources); motivations (beliefs about capabilities and consequences, identity, optimism, goals, intentions, emotion, reinforcement)* |
| 65-80mins | **Role of digital technologies**   1. Discuss how you or the person you support uses digital technologies (e.g. mobile apps, telehealth and web-based applications) for managing their LTC 2. Discuss how digital technologies might help with managing a LTC   *Prompts: acceptability, barriers, email and text communication, monitoring symptoms / activity* |
| 80-90mins | **CLOSE SESSION**   - Summarise key points - Remind participants about confidentiality - Thank participants |
