# Supplementary figures and images for: The lived experience of severe mental illness and long-term conditions: a qualitative exploration of service user, carer, and healthcare professional perspectives on self-managing co-existing mental and physical conditions
Source: BMC Psychiatry. 2022 Jul 19;22:479. doi: 10.1186/s12888-022-04117-5 (PMC9295434; doi:10.1186/s12888-022-04117-5)

**Appendix B. Visual storyboard**
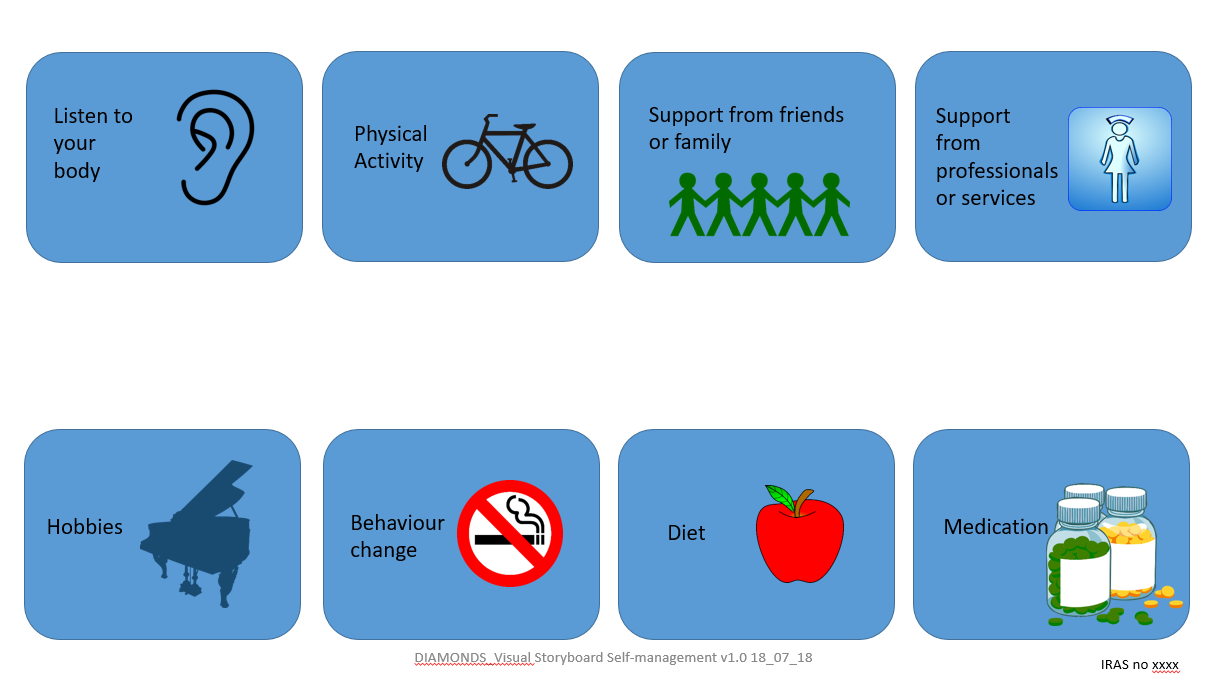

Supplement: Supplementary file 2 — Additional file 2. [file 12888_2022_4117_MOESM2_ESM.docx]
