# Supplementary material for: The lived experience of severe mental illness and long-term conditions: a qualitative exploration of service user, carer, and healthcare professional perspectives on self-managing co-existing mental and physical conditions
Source: BMC Psychiatry. 2022 Jul 19;22:479. doi: 10.1186/s12888-022-04117-5 (PMC9295434; doi:10.1186/s12888-022-04117-5)
Supplement: Supplementary file 3 — Additional file 3. [file 12888_2022_4117_MOESM3_ESM.docx]

**Appendix C. QUEST Participant Interviews Coding Framework**

QUEST Participant Interviews

Coding framework V2

N.B: please code negative responses as well, e.g. where someone says something has no impact, or no effect etc. Helps give the full picture.

| Name | Description |
| --- | --- |
| **Demographics and context** |  |
| Age |  |
| Caring responsibilities | Anyone person is responsible for (children, parents etc.) |
| Current medication | Medications being taken |
| Diagnoses |  |
| Ethnicity |  |
| Family details | What immediate family does the person mention (if any) |
| Family health history |  |
| Hobbies and interests |  |
| Home and local environment |  |
| Lifestyle |  |
| Mobility |  |
| Money and income |  |
| Past trauma |  |
| Religious beliefs |  |
| **Health education and knowledge and training** |  |
| Access to education and knowledge |  |
| Barriers to education and knowledge |  |
| Experience of formal education courses |  |
| Impact of education and knowledge | Positive or negative |
| Knowledge of LTC(s) | Any knowledge, or gaps in knowledge demonstrated of their LTC |
| Knowledge of LTC(s) self-management | As above, for diabetes management |
| Sources of information | All formal and informal sources of information |
| **Lived experience of LTC(s)** |  |
| Burden of LTC(s) | Experiences that relate to the strain of living with an LTC, including emotional, physical, and social. |
| Symptoms and complications of LTC(s) |  |
| Impact of LTC on self-management(s) |  |
| Sources and type of support for LTC(s) |  |
| Duration of diagnosis and prognosis | When was the LTC diagnosed.  Any other comments on timings and prognosis of the LTC. |
| Experience /perception of LTC(s) | General experiences (both positive and negative), or more general thoughts and perceptions of the LTC(s) |
| Good days and bad days |  |
| Perceived causes |  |
| Stigma and discrimination | Any experiences of stigma or discrimination (including imagined/perceived) as a result of their LTC(s) |
| **Experience of mental health care** | Any type, both specialist and primary |
| Access to care |  |
| Barriers to care |  |
| Changes to care |  |
| Experience of medication |  |
| Experience of medication side-effects |  |
| Opinions of psychiatric medication |  |
| Impact of care | Positive and negative |
| Involvement in care decisions | Involvement, or not, in care decisions |
| Opinions on health care |  |
| Personal experiences of mental health care | Any other experiences that don’t fit the specific categories. |
| Power dynamics | Power dynamics between patient and staff |
| Relationships with mental health professionals | Positive or negative description of relationships with mental health professionals |
| Timing of care received |  |
| Understanding of care received |  |
| Wishes for and thoughts on improvements |  |
| Worries about health care |  |
| **Lived experience of mental illness** |  |
| Behaviours associated with mental illness | Behaviours that aren’t necessarily symptoms, e.g. over-spending due to impulse control. |
| Burden of mental illness |  |
| Coping mechanisms | Positive and negative (e.g. mindfulness, self-harm) |
| Crisis points |  |
| Current state of mental health | Perceived and actual |
| Disclosing mental illness | Disclosing, or choosing not to |
| Effect of outside influences | E.g. weather, holidays etc. |
| Sources of support for mental health |  |
| Good days and bad days |  |
| Impact of mental illness | Positive and negative |
| Not feeling in control |  |
| Others' opinions and perceptions | Real/experienced and perceived |
| Perceived causes |  |
| Perceptions of mental illness | General thoughts on mental illness, not necessarily own condition |
| Stigma and discrimination | Experienced or imagined |
| Symptoms of mental illness |  |
| Understanding and perceptions of own illness | Any thoughts about own illness specifically |
| **Experience of physical health care for LTCs** |  |
| Access to care |  |
| Barriers to care |  |
| Experience of medication |  |
| Experience of treatment for LTCs |  |
| Involvement in care decisions | Involvement, or not, in decisions |
| Opinions on care |  |
| Personal experiences of physical health care | Any experiences which don’t fit into other specific categories |
| Wishes and thoughts for improvement |  |
| **Multi-morbidity and Interaction of LTC and SMI** |  |
| Recognition of multiple LTCs | Code self-reported multiple LTCs. |
| LTC specific interaction with SMI |  |
| Descriptions of interactions | Interactions between diabetes and SMI. Include ‘no interaction’ comments. |
| LTC takes priority |  |
| Impact of LTC(s) on mental health | Including impact on self-management, care etc. as well as symptoms |
| Impact of mental health on LTC(s) | As above |
| Interactions between mental and physical care | Do staff discuss care, is there crossover between care etc.? |
| Mental health takes priority |  |
| **Informal support and social contact** |  |
| Sources of support |  |
| Barriers to support |  |
| Access to social support | Accessibility of social support (e.g. local neighbourhood) |
| Types of support |  |
| Impact of support | Positive and negative |
| Loss of support |  |
| Rejecting support | Experiences of and reasons for rejecting any support |
| **Digital technology and self-management** |  |
| Access to digital technology |  |
| Barriers to digital technology use |  |
| Types of digital technology |  |
| Use of digital technology in self-management | Code the ways participants have used digital technology to support their self-management |
| Impact of digital technology | Positive and negative |
| **Self Management** |  |
| Barriers to self-management |  |
| Enablers of self-management |  |
| Feeling in control | Or not feeling in control of self-management |
| Impact of self-management | Positive and negative |
| Poor self-management | Instances of poor self-management, e.g. eating a lot of sugar |
| Self-management behaviours | Instances of self-management behaviours, e.g. exercise |
| Self-management success | Any successes, e.g. weight loss, joining gym |
| Support for self-management | Any support available for self-management |
| Tools for self-management | e.g. blood sugar monitor, dosette box for tablets |
| Worries about self-management |  |
| **Unsure** | Anything that doesn’t fit the categories. |
